# Comparative Analysis of Nutritional Advice and a Combined Approach for Addressing Impending Stunting in Infants: A Clinical Trial

**DOI:** 10.3390/nu16172832

**Published:** 2024-08-24

**Authors:** Conny Tanjung, Bahrul Fikri, Titis Prawitasari, Nasrum Massi, Andi Alfian Zainuddin, Aidah Juliaty, Dwi Sora Yullyana, Sarah Dwitya, Naoki Shimojo, Hiroshi Ohno, Berthold Koletzko

**Affiliations:** 1 Post Graduate School, Hasanuddin University, Makassar 90245, Indonesia; 2 Department of Pediatrics, Faculty of Medicine, Hasanuddin University, Makassar 90245, Indonesia; bahrulfikriyahya@gmail.com (B.F.); aidah_juliaty@yahoo.com (A.J.); 3 Department of Child Health, Dr. Cipto Mangunkusumo National Central Hospital, Faculty of Medicine, Universitas Indonesia, Jakarta 10430, Indonesia; tprawitasari@yahoo.com; 4 Department of Microbiology, Faculty of Medicine, Hasanuddin University, Makassar 90245, Indonesia; nasrumm2000@yahoo.com; 5 Public Health and Community Medicine Department, Faculty of Medicine, Hasanuddin University, Makassar 90245, Indonesia; a.alfian@med.unhas.ac.id; 6 Institute of Research and Community, Microbiome Research Division, Hasanuddin University, Makassar 90245, Indonesia; yullyana.sora@gmail.com; 7 Telkom Indonesia Health Foundation, Bandung 40133, Indonesia; sarahdwitya@ymail.com; 8 Center for Preventive Medical Sciences, Chiba University, Chiba 263-8522, Japan; shimojo@faculty.chiba-u.jp; 9 Laboratory for Intestinal Ecosystem, RIKEN Center for Integrative Medical Sciences (IMS), Yokohama 230-0045, Japan; hiroshi.ohno@riken.jp; 10 Department of Pediatrics, Dr. von Hauner Children’s Hospital, LMU-Ludwig Maximilians Universität Munich, 80337 Munich, Germany; berthold.koletzko@med.uni-muenchen.de

**Keywords:** weight faltering, oral nutritional supplements, nutritional advice, weight increment

## Abstract

Weight faltering (WF) has been associated with stunting and with long-term adverse consequences for health and development. Nutritional care for managing WF may consist of giving nutritional advice (NA) and/or provision of oral nutrition supplements (ONSs). In this study, we aimed to evaluate practical management options in the community for infants with WF aged 6–12 months. This nonrandomized clinical trial was conducted in the community of Makassar, South Sulawesi, from March 2022 to March 2023. A total of 1013 infants were enrolled for screening. Anthropometric measures were performed in 913 infants, of which 170 showed WF below the 15th percentile of the WHO weight increment table without stunting. Infants with a weight increment below P5th were assigned to receive NA plus ONS, while infants between P5th and below P15th were assigned to receive only NA. At the second and third months, ONSs were administered to WF infants who were below P15th. One month after the intervention, 87/105 infants in the NA-plus-ONS group (82.8%) and 52/65 infants in the NA-only group (80%) were no longer WF. After 3 months, infants in the NA-plus-ONS group achieved greater weight gain than infants in the NA group (264.1 g vs. 137.4 g, *p* < 0.001) as well as greater length gain (2.35 cm vs. 2.14 cm, *p* < 0.001). WF management should be started at below P15th to achieve a better result. Infants with greater nutritional deficits should be assigned to receive the combination of NA plus ONSs to achieve a higher rate of resolution of growth.

## 1. Introduction

Child undernutrition and disadvantaged household environments contribute to deficits in children’s development and health and productivity in adulthood [1]. Inadequate weight gain over prolonged time periods is associated with reduced linear growth and stunting [2]. Onyango et al. found that the prevention of weight loss below the 15th percentile of the WHO weight increment table is associated with 34% less stunting at the age of one year and 24% less stunting at the age of two years. The selected threshold was applied in a cohort of Bangladeshi infants to assess its predictive value for stunting at the ages of 12 months and 24 months [3].

Pediatric nutritional care (PNC) is a key element in managing WF [4,5]. The proposed steps of PNC include an evaluation of the patient’s medical and nutritional condition, a determination of nutritional needs, a selection of appropriate foods and method of delivery, and monitoring any potential side effects or acceptance issues [6]. ONSs are considered as an option when available regular food does not provide adequate nutrition. ONSs are products that contain multiple nutrients and are consumed to increase the patient’s energy and nutrient intake [7]. In different ONS products, the density of energy and nutrients varies. Some ONSs are nutritionally complete and can cover the total nutrient needs of an infant or child, e.g., if entirely tube-fed, while others designed to be used only to supplement extra energy and selected nutrients, e.g., protein, are not nutritionally complete, and should only be used as supplementary sources of nutrition in addition to other dietary products [8,9].

Managing WF in infants aged 6 to 12 months can be challenging, particularly when resources and qualified medical staff, such as pediatricians, general practitioners, midwives, nutritionists, or nurses, are limited. This study aims to explore practical, feasible, and effective interventions for WF infants in the setting of disadvantaged communities in Indonesia NA alone or NA in combination with the use of ONS.

## 2. Materials and Methods

### 2.1. Subjects

Participating infants and their families were recruited by the staff of 30 primary health facilities in Makassar, South Sulawesi, Indonesia. Eligible for inclusion were infants aged 6–12 months at the time of study entry who met the criteria shown below and whose parents provided written informed consent. Participants were included between March 2022 and March 2023.

Inclusion Criteria:

1. Infants aged 6–12 months with WF, defined as weight increments < P15th of the WHO weight increment table [10];
2. Growth chart available for monitoring (weight, length, and head circumference measured at least once at birth);
3. Available height and weight data from both father and mother;
4. A parent (either mother or father) agreed to participate in the study and signed their informed consent.

Exclusion Criteria:

1. Subjects with a length-for-age *z*-score (HAZ) below 2 SD;
2. Severe acute malnutrition;
3. Presence of cow’s milk allergy;
4. Presence of lactose intolerance;
5. Presence of galactosemia;
6. Major congenital anomaly, severe stunting at birth (newborns whose length-for-gestational age was below 10th percentile), thyroid disorder, major gastrointestinal disease, or other severe diseases, e.g., pneumonia or dehydration;
7. Conditions that require special diets, e.g., major renal or hepatic dysfunctions;
8. Conditions that influence nutritional status, e.g., moderate to severe dehydration, edema, organomegaly;
9. Infants with relative WF but a body weight above the median weight for length (considering that they may become overweight);
10. History of a low birth weight (less than 2500 g).
11. History of premature birth (born after a period of pregnancy of less than 37 weeks).

### 2.2. Study Design

The study was initially planned as a randomized controlled trial (RCT) among weight-faltering infants (with a weight increment below the 15th percentile). However, most infants identified during screening had weight increments below the 5th percentile (P5). According to Indonesian Health Ministry Regulation No. 29 (2019), there is a need to support those infants who have a weight increment below P5 with the ONS. Therefore, it was not ethical for us to conduct an RCT.

The study compared two groups of subjects assigned to two different treatments based on the degree of WF, with a follow-up period of 3 months. The ONSs were given to parents of eligible infants recruited into the study and assigned to the ONS group. All participants in this study were treated in accordance with the Declaration of Helsinki. Ethical review was performed by The Permanent Medical Research Ethics Committee in Medicine and Health/Faculty of Medicine Hasanuddin University. No. 98/UN 4.6.4.5.31/PP36/2022 and extended with No. 56/UN 4.6.4.5.31 /PP36/2O23. This study has been registered to clinicaltrial.gov (NCT05393934).

In the first phase of the intervention (1 month), infants with a weight increment below P5th at baseline were assigned to receive NA plus ONSs. The intervention study product was a powder-based infant formula with high nutrient density, with an energy density of 100 kcal/100 mL, and a protein to energy ratio of 10%, commercially available as SGM Ananda Gain 100^®^, developed by PT Sarihusada Generasi Mahardhika, in Jakarta, Indonesia. Infants with weight increments between P5th and below P15th were assigned to only NA. 

**Figure 1:**
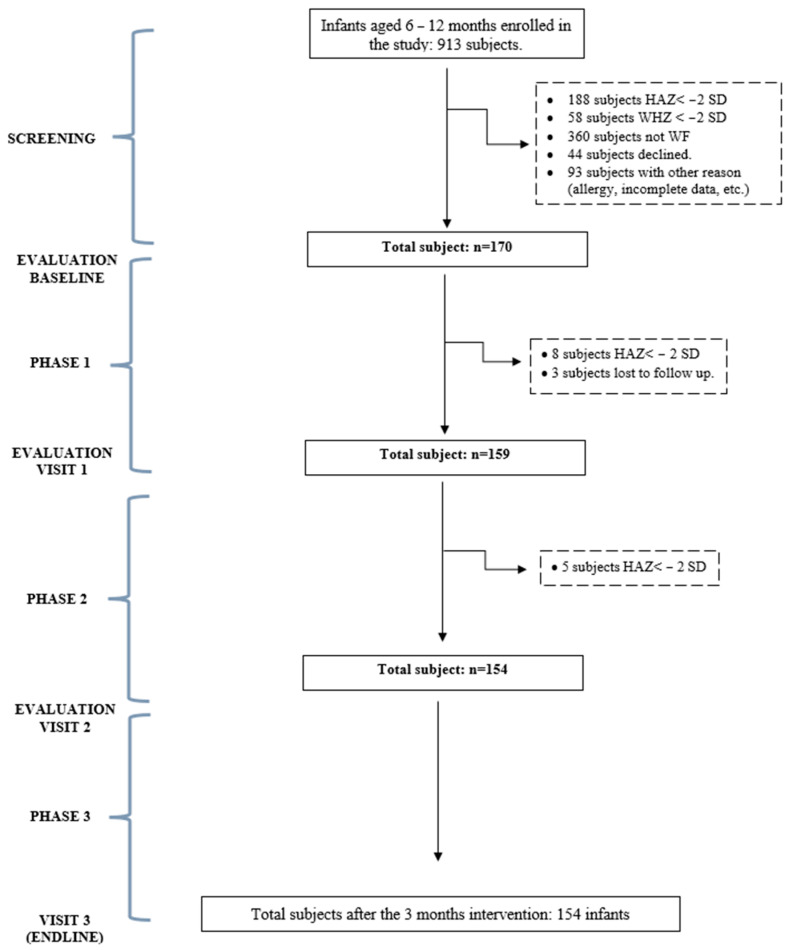
Subject selection flow. HAZ = height-for-age-*z* score; WHZ = weight-for-height *z*-score; WF = weight faltering.

In the second phase (second and third months) of the intervention, once an infant had achieved an adequate WI above the P15th of the WHO weight increment table, the ONS provision was discontinued. If the subject initially had adequate weight increment, but experienced WF again, the subject was given NA plus ONSs. Figure 1 shows the subject selection and follow-up flow.

Nutritional advice was given to parents regarding the type, composition of macro- and micro-nutrients, and amount of food required in order to increase their infants’ weights accordingly. Nutritional requirements were customized for each subject based on an estimated calorie deficit, calculated according to energy needs at their ideal weight according to length. Families were advised to provide the recommended amount of ONS divided into 2–3 portions over 1 day. Parents were educated on ONS preparations, including good hygiene standards, the right amount of ONSs, and dissolution.

Study visits were conducted at the time of study enrolment (baseline) and after approximately one, two, and three months, respectively. Routine clinical care was also offered for all participating infants, involving physical examination, anthropometric measurement, and health and nutritional education (e.g., infant and young child feeding practices, personal hygiene, and hand washing). At every visit, parents were asked about the type and amount of food and milk consumed (a 72 h food recall); breastfeeding frequency; eating patterns; sleeping patterns; the incidence of illnesses such as fever, cough, runny nose, diarrhea, and/or vomiting; along with the infants’ general conditions as a whole. The research team also remeasured body weight, length, and head circumference. Aside from dietary analyses, product acceptance questionnaires for the ONS products were assessed during those visits. In addition, the field assistant monitored the total volume of ONSs consumed through weekly phone calls. The occurrence of adverse events and medication use was recorded throughout the study period. Trained medical doctors and nutritionists conducted all of the monitoring. All subjects were provided with insurance from the Social Security Agency for Health for up to one month after the study was completed.

## 3. Results

A total of 1031 infants from 30 primary health facilities in Makassar, South Sulawesi, were enrolled for screening, and anthropometric measures were performed in 913 infants aged 6–12 months old, of which 170 showed WF below the 15th WHO percentile of weight increment (WI) without stunting (Figure 1). One hundred and five infants had weight increments below the fifth percentile of the weight increment table (<P5th) and received NA plus ONSs according to their daily caloric needs based on their ideal body weight determined by their length. The data demonstrated a significant increase in weight gain of 0.423 kg ((95% CI 0.234, 0.613), *p* < 0.001) and height gain of 0.417 cm ((0.059, 0.776), *p* = 0.022) within the ONS intervention group. And to fulfil this energy deficit in all subjects below the P5th group, the mean amount of ONS given to infants was 77.38 mL per day. Sixty-five infants between the P5th and below the P15th percentile were assigned to receive NA only. All subjects were followed for three months.

The characteristics of the infants were similar in both groups. Most infants (when they were younger than 6 months) were exclusively breastfed (71.4% in NA-plus-ONS group and 80.0% in NA-only group). Maternal education, which also played an important role in the continuity of this study, did not differ much. Of the mothers in the NA-plus-ONS group, 65.7% received an upper-secondary education, while 66.2% of mothers in the NA-only group received an upper-secondary education. A majority of infants have received complementary feeding. Specifically, 91.4% in the NA-plus-ONS group and 92.3% of the infants in the NA-only group. The history of infant illness before intervention in the NA-plus-ONS group was 59.0%, while in the NA-only group, it was 52.3%.

After one month of intervention, weight increased significantly in both groups (Table 1). After two and three months, infants assigned to receive NA plus ONS showed a significantly higher increment of body weight, length, and BMI than the group assigned to NA only. Finally, after 3 months, infants in the NA-plus-ONS group were found to achieve greater weight gain than infants in the NA-only group (264.1 g vs. 137.4 g, *p* < 0.001).

**Figure 2:**
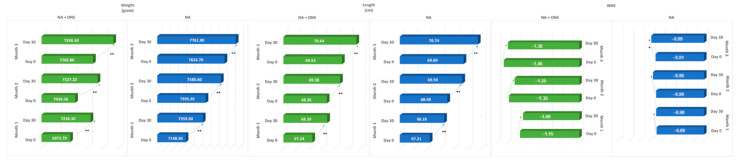
The changes in weight, length, and WHZ within groups during the intervention. Notes: ONS = oral nutritional supplement; NA = nutritional advice; phase one = consumption of ONSs starts from <P5, and NA only for infants P5–<P15; phases two and three = consumption of ONSs starts from <P15 (early intervention); P = percentile; * *p*-value < 0.05, ** *p*-value < 0.001.

This study found that the nutrition status condition improved within the group of infants assigned to receive NA plus ONS, which is illustrated by significant weight gain ranging from 263.5 to 298 g throughout the study (from phases 1 to 3, *p* < 0.05). Additionally, a significant height increase ranging from 0.97–1.15 cm was observed. The increases in weight and length also reflected the improvement in the WHZ scores, as can be seen in Figure 2.

Grouping the subjects according to their response to the intervention, we could see that during the first month of the intervention, 87 of 105 infants (82.8%) assigned to receive NA plus ONSs gained weight greater than the 15th percentile of the WHO weight increment table, and 75 (71.4%) were no longer WF. In the group assigned to NA, 52 of 65 infants (80%) gained weight greater than the P15th. Fewer infants showed weight loss in the NA-plus-ONS group than in the NA-only group (1.9% vs. 9.2%, *p* = 0.001).

In the second phase of the intervention (second month) when the subjects were divided into WF and non-WF, 85.7% of the NA-plus-ONS group had achieved a weight increment above the P15th (30 subjects). In comparison, 5.7% (two subjects) were still in the WF condition. On the other hand, 76.6% (95 subjects) in the NA-only group maintained the non-WF condition. Regarding the subjects that experienced weight loss, only 2.9% (1 subject) were in the NA-plus-ONS group, while 23.4% (29 subjects) were in the NA-only group.

**Figure 3:**
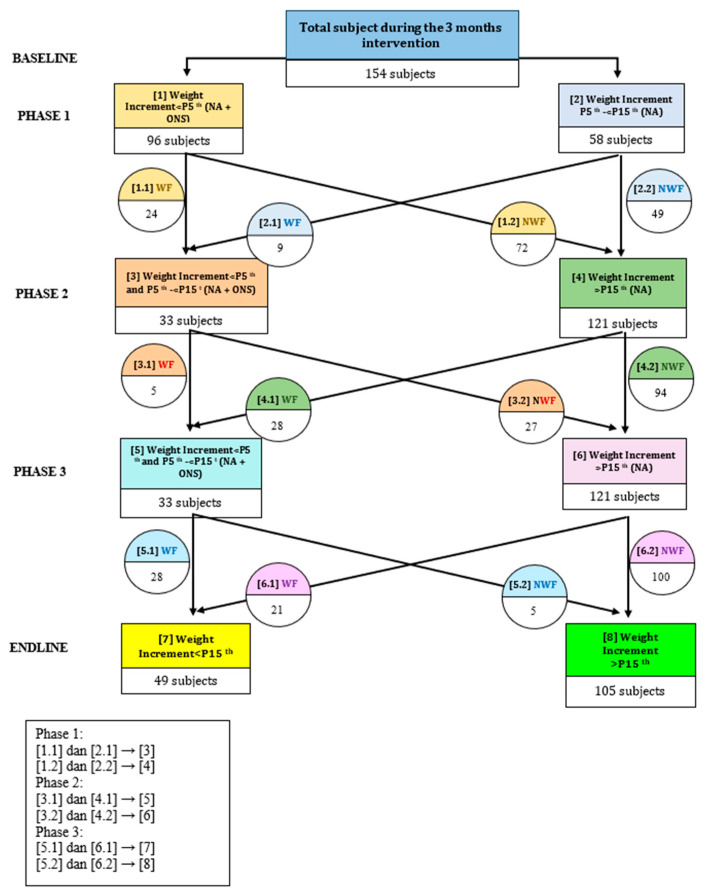
Changes in non-WF and WF condition after each phase of the intervention, excluding those who were dropped out in the study. In phase one, those with a weight increment < P5th were assigned NA plus ONSs and those with a weight increment P5th to < P15th were assigned NA only; in phases two and three, only those who still had a weight increment < P15th (still WF) were assigned NA plus ONSs.

As can be seen in Figure 3, in the third month of this study, 79.4% of infants (27 subjects) from the NA-plus-ONS group achieved a weight increment above the P15th, and only 2.9% (1 subject) were still in the WF condition. Conversely, in the NA-only group, no infants experienced an increased percentile increment, but 76.5% (91 subjects) managed to maintain the non-WF condition, while as many as 23.5% (31 subjects) experienced WF again. Data from the study monitoring found several causes of decreasing body weight, namely frequent illnesses such as respiratory infections for more than a week, diarrhea, and parental non-compliance with the recommended nutritional advice.

Regarding the sustainability of the non-WF condition from the phase one intervention, 52.1% subjects that directly received NA plus ONS at phase one were still in the non-WF condition after 3 months of follow-up without ONS. Furthermore, 37.9% of subjects between P5th and < P15th could still be managed with NA alone until 3 months of follow-up. 

In phase two, almost all subjects below the 15th percentile who received NA plus ONSs were no longer in the weight-faltering (WF) condition after 2 months. One subject remained in WF due to frequent illness. Additionally, five infants with weight increments below the fifth percentile at baseline experienced adverse events, but no serious adverse events (SAEs) occurred.

## 4. Discussion

Recent studies have underscored the importance of a comprehensive approach to managing weight faltering in children, which extends beyond the current focus of the World Health Organization (WHO) guidelines on acute malnutrition [11]. While the WHO guidelines provide a robust framework for addressing acute malnutrition, they do not specifically address weight faltering, a condition that may precede or occur independently of acute malnutrition. Weight faltering, characterized by slow weight gain or weight loss, can have significant long-term impacts on a child’s growth and development. Therefore, it is crucial to integrate insights from this study into weight-faltering management strategies. This would involve tailoring interventions to individual needs by considering factors such as dietary intake, feeding practices, and underlying health conditions. With these tailored interventions, a more holistic approach to child nutrition and health can be ensured, which ultimately improves outcomes for children worldwide.

Weight-faltering management is the cornerstone for the prevention of many nutritional disorders and can help ensure children’s right to growth and well-being. The responsibility for managing weight faltering is not only on parents and health professionals, but also involves all parts of the community throughout the country [12]. Providing a good nutritional intervention to WF children has improved nutrient intake and driven catch-up growth, leading to better outcomes and preventing further issues [6,12,13,14]. There are several ways of handing the WF condition, such as administering NA only or NA plus ONS. Most studies on nutritional interventions utilizing ONS focus on children older than 1 year old [15,16,17]. To the best of our knowledge, there is a scarcity of intervention studies focusing on infants, especially in the community. 

Our research used two types of interventions, namely administering NA only or NA plus ONS. In the NA-only group, we provided simple food composition advice and instructions, adjusting for the socio-economic condition of the subject, while promoting the local food policy in accordance with the agreement with the government. The recommended food ingredients were readily available, with quantities that were financially affordable and familiar to families, such as egg, chicken, liver, fish, beef, coconut milk, oil, cheese, potatoes, and rice. In this study, we found that the mean amount of ONSs needed for the below P5th infants was only 77.38 mL per day. This amount was well tolerated. There were no complaints from the parents in accepting the ONS due to its positive results and the moderate amount that called for no change to their infants’ diets.

In phase one (first month) of the intervention, both intervention groups showed positive results, but could not maintain a sustained adequate weight increment as some infants entered into WF again. In a prior study encompassing children under 5 years old in a poor urban setting, improvements were observed in anthropometric parameters after authors administered a nutritional formula for 90 days [18].

The findings after three months showed that infants in the NA-plus-ONS group showed more promising results compared to the NA-only group, as reflected by the change in mean *z*-score between the three main anthropometric measurements evaluated: length-for-age, weight-for-length, and weight-for-age *z*-scores. The infants who received NA plus ONSs monthly experienced a stable increase. Meanwhile, infants who received NA only at the length-for-age and weight-for-length *z*-score experienced extreme increases and decreases in *z*-score values. 

Looking deeper into the sustainability of the non-WF condition from phase one of the intervention, it is seen that more than half of the subjects that received NA plus ONSs at the beginning did not need ONSs by the end of the study. For subjects that were in less-severe condition that received NA only, more than 1/3 of them still could be maintained by NA only. From the second phase of the intervention, it is seen that almost all WF subjects who received NA plus ONSs managed to be in the non-WF category by the end of the study. 

Understanding the eligibility criteria of the weight-faltering infants in the study allows for an appropriate generalization of the results. Based on these data, it becomes necessary to consider initiating an intervention that provides tailor-made nutritional education to each individual in conditions below the P15th percentile. Nevertheless, if infants have fallen below the P5th percentile, ONSs should be immediately administered as a complimentary addition to regular nutrition education, which can accelerate the improvement of nutritional status in a shorter time. This might suggest a cost-effective intervention. 

The superior use of ONSs for malnutrition intervention, supported by a recent meta-analysis, revealed notable advancements in weight and height among children who underwent the ONS intervention compared to the control group. The data demonstrated a significant increase in weight gain of 0.423 kg ([95% CI 0.234, 0.613], *p* < 0.001) and height gain of 0.417 cm ([0.059, 0.776], *p* = 0.022) within the ONS intervention group. This positive effect on weight gain was evident 7–10 days after the intervention commenced. The longitudinal analyses, conducted with repeated measurements at 30-, 60-, and 90-day intervals, consistently highlighted substantial improvements in weight parameters from the 30-day mark onward (*p* < 0.001). Additionally, a tendency toward enhanced height gains emerged at the 90-day mark (*p* = 0.056) [9,19]. On the other hand, there is a need to note that worse nutrition among those that underwent the ONS intervention (vs. those in the control group) may be one of the contributing factors. 

While we have highlighted the potential benefits of combining ONSs with NA in the community, several limitations of this study should be noted. First, the study duration of three months was insufficient to capture the long-term effects of the intervention on growth patterns beyond this initial period. Second, during recruitment, we encountered many infants who were below P5 or already stunted; therefore, this study cannot be conducted using a randomized clinical trial methodology, as consideration must be given to providing immediate interventions to infants with weight gain below P5 who are prone to stunting. This is supported by the finding in this study that all AEs were experienced by those who had weight gain below P5 at baseline. Third, the interpretation of results from this study needs to consider the allowance of crossing one intervention group with another. Fourth, there were no individual laboratory tests for all subjects. 

As Indonesia holds a concerning stunting rate above the world average with no precise WF rate and the highest number of vulnerable age groups to WF, the implications of this study will add to the wealth of data in Indonesia and to our knowledge, this study is the first study on the intervention of infants with WF condition involving subjects at an early age.

Further studies involving a more comprehensive examination of external factors to understand their contribution to growth outcome, with a period beyond 3 months or following up after a certain period (e.g., after 18 and 24 months of the completed intervention), are needed to assess the effect of the intervention on weight-faltering infants’ growth either as individuals or as a whole, including the somatic outcomes. Exploring the mechanisms by which the integration of NA plus ONSs affects infant growth could lead to more effective strategies.

Broadening the demographic scope of the study and conducting a comparative analysis of various interventions can help identify appropriate approaches for specific infant populations. Collaborations involving health professionals, nutritionists, psychologists, and social scientists can offer multifaceted insights. Lastly, incorporating longitudinal follow-ups can provide valuable insights into the enduring effects of early interventions on the subsequent growth and developmental outcomes.

## 5. Conclusions

In this study, we included 6–12-month-old infants in community-based settings who suffered from WF using the weight increment from the WHO table. We compared the weight increment of above 5th and below the 15th percentile subjects that were given NA only with the NA plus ONS group in infants with a weight increment below the 5th percentile. After one month of the intervention, both groups showed an improved weight increment, suggesting that the use of below P15th should call for action for the correction of WF.

This study shows that starting the intervention at below P15th could offer a better result for the WF infants. Integrating a small amount of ONSs into the NA regimen for the WF infants below P5th demonstrates a good and sustainable result with high feasibility and acceptance. It is important to note that ongoing nutrition interventions with accompaniment from cadres and health professionals are needed to maintain growth and overall well-being among infants facing growth challenges.

## Figures and Tables

**Table 1:** Anthropometric changes during intervention.

Phase	Variable	N	NA + ONS (Mean ± SD)		*p*-Value *	N	NA (Mean ± SD)		*p*-Value *
			Screening	Visit 1			Screening	Visit 1	
1	Weight (gr)	105	6972.70 ± 641.55	7236.50 ± 673.05	<0.001	65	7148.30 ± 676.65	7359.00 ± 720.63	<0.001
	Length (cm)	105	67.24 ± 2.23	68.39 ± 2.23	<0.001	65	67.21 ± 2.20	68.26 ± 2.21	<0.001
	BMI (kg/m^2^)	105	15.40 ± 0.82	15.45 ± 0.92	0.383	65	15.80 ± 0.99	15.77 ± 1.02	0.611
	HAZ	105	−0.86 ± 0.66	−0.98 ± 0.67	<0.001	65	−0.97 ± 0.64	−1.11 ± 0.66	<0.001
	WHZ	105	−1.15 ± 0.60	−1.09 ± 0.67	0.198	65	−0.89 ± 0.68	−0.90 ± 0.69	0.974
	WAZ	105	−1.39 ± 0.59	−1.40 ± 0.65	0.761	65	−1.21 ± 0.59	−1.26 ± 0.62	0.34
	Weight Increment (gr)	105	Not Available	263.77 + 276.28		65	Not Available	210.85 ± 216.26	
	**Variable**	**N**	**NA + ONS (Mean ± SD)**		*p*-Value *	**N**	**NA (Mean ± SD)**		*p*-Value *
			**Visit 1**	**Visit 2**			**Visit 1**	**Visit 2**	
2	Weight (gr)	35	7059.60 ± 539.50	7357.60 ± 573.78	<0.001	124	7393.20 ± 682.48	7580.60 ± 703.32	<0.001
	Length (cm)	35	68.42 ± 1.90	69.39 ± 1.86	<0.001	124	68.49 ± 2.272	69.588 ± 2.27	<0.001
	BMI (kg/m^2^)	35	15.06 ± 0.68	15.27 ± 0.79	0.014	124	15.74 ± 0.96	15.63 ± 0.99	0.025
	HAZ	35	−1.10 ± 0.68	−1.17 ± 0.72	0.114	124	−0.94 ± 0.62	−1.02 ± 0.64	0.002
	WHZ	35	−1.35 ± 0.57	−1.21 ± 0.60	0.072	124	−0.90 ± 0.65	−0.96 ± 0.69	0.094
	WAZ	35	−1.59 ± 0.62	−1.50 ± 0.66	0.218	124	−1.21 ± 0.56	−1.25 ± 0.59	0.165
	Weight Increment (gr)	35	−36.54 ± 174.24	300.00 ± 225.46	<0.001	124	340.73 ± 207.58	226.62 ± 236.49	<0.001
	**Variable**	**N**	**NA + ONS (Mean ± SD)**		*p*-Value *	**N**	**NA (Mean ± SD)**		*p*-Value *
			**Visit 2**	**Visit 3**			**Visit 2**	**Visit 3**	
3	Weight (gr)	34	7266.80 ± 665.97	7530.30 ± 693.62	<0.001	120	7624.70 ± 671.06	7761.90 ± 626.34	<0.001
	Length (cm)	34	69.53 ± 2.43	70.64 ± 2.35	<0.001	120	69.69 ± 2.01	70.74 ± 2.14	<0.001
	BMI (kg/m^2^)	34	15.00 ± 0.71	15.06 ± 0.66	0.523	120	15.68 ± 1.00	15.50 ± 0.93	0.002
	HAZ	34	−1.13 ± 0.68	−1.12 ± 0.73	0.799	120	−0.97 ± 0.61	−0.99 ± 0.66	0.383
	WHZ	34	−1.46 ± 0.53	−1.38 ± 0.49	0.305	120	−0.91 ± 0.69	−0.99 ± 0.64	0.038
	WAZ	34	−1.66 ± 0.55	−1.58 ± 0.53	0.227	120	−1.18 ± 0.59	−1.23 ± 0.59	0.088
	Weight Increment (gr)	34	51.32 ± 289.00	264.12 ± 271.07	0.014	120	285.55 ± 177.58	137.35 ± 278.18	<0.001

NA = Nutritional Advice; ONS = Oral Nutritional Supplement; BMI = Body Mass Index; HAZ = Height-for Age-Z score; WHZ = weight-for-height Z-scores; WAZ = Weight-for-Age-*Z* score; * *p* Value reported refers to *t* test between levels the two visits within the same group (the 2 columns in the left side).

## Data Availability

The original contributions presented in the study are included in the article, further inquiries can be directed to the corresponding author.
